# Role of “Sural Sparing” Pattern (Absent/Abnormal Median and Ulnar with Present Sural SNAP) Compared to Absent/Abnormal Median or Ulnar with Normal Sural SNAP in Acute Inflammatory Demyelinating Polyneuropathy

**DOI:** 10.3389/fneur.2017.00512

**Published:** 2017-09-29

**Authors:** Spurthi Sunil Surpur, Raghav Govindarajan

**Affiliations:** ^1^University of Missouri, Columbia, MO, United States; ^2^Department of Neurology, University of Missouri, Columbia, MO, United States

**Keywords:** sural sparing, acute inflammatory demyelinating polyneuropathy, chronic inflammatory demyelinating polyneuropathy, axonopathy, diabetic neuropathy

## Abstract

**Background:**

Sural sparing defined as absent/abnormal median sensory nerve action potential (SNAP) amplitude or absent/abnormal ulnar SNAP amplitude with a normal sural SNAP amplitude is thought to be a marker for inflammatory demyelinating polyneuropathies.

**Objective:**

If sural sparing pattern specifically defined as absent/abnormal median and ulnar SNAP amplitude with normal sural SNAP amplitude (AMUNS) is sensitive and specific when compared with either absent/abnormal median and normal sural (AMNS) or absent/abnormal ulnar and normal sural (AUNS) for acute inflammatory demyelinating polyneuropathy (AIDP), chronic inflammatory demyelinating polyneuropathy (CIDP), select non-diabetic axonopathies (AXPs), and diabetic neuropathies (DNs).

**Method:**

Retrospective analysis from 2001 to 2010 on all newly diagnosed AIDP, CIDP, select non-diabetic AXP, and DN.

**Results:**

There were 20 AIDP and 23 CIDP. Twenty AXP and 50 DN patients between 2009 and 2010 were included as controls. AMUNS was seen in 65% of AIDP, 39% CIDP compared with 10% of AXP and 6% for DN with sensitivity of 51%, specificity of 92%, whereas the specificity of AMNS/AUNS was 73% and its sensitivity was 58%. If a patient has AMUNS they are >12 times more likely to have AIDP (*p* < 0.001).

**Conclusion:**

Sural sparing is highly specific but not sensitive when compared with either AMNS or AUNS in AIDP but does not add to sensitivity or specificity in CIDP.

## Introduction

A common scenario where nerve conduction studies are of particular use is in differentiating primary axonal from primary demyelinating neuropathies (DMNs).

Sensory electrodiagnostic studies although routinely performed are rarely, if at all used in this setting. In fact, none of the major diagnostic criterions for acute inflammatory demyelinating polyneuropathy (AIDP) (1, 2) and chronic inflammatory demyelinating polyneuropathy (CIDP) (3–5) include sensory EDX parameters except the American Academy of Neurology *Ad Hoc* subcommittee Criteria which recognizes sensory conduction velocity less than 80% of the lower limit as being supportive for CIDP (6).

Sural sparing defined variously as normal or relatively preserved sural sensory nerve action potential (SNAP) amplitude with abnormal/absent median SNAP amplitude and/or abnormal/absent ulnar SNAP amplitude and/or abnormal/absent radial SNAP amplitude (7–11) has been known to be a highly specific marker for DMNs.

Previous studies that looked into sensitivity and specificity of sural sparing pattern in either AIDP (7, 8) or CIDP (10, 11) lacked a uniform definition for sural sparing and in many cases patients were already on treatment thus potentially confounding the EDX results.

Further most studies use either an abnormal/absent median or abnormal/absent ulnar with normal sural in defining sural sparing. Both median and ulnar nerves are commonly susceptible to compression and thus there is a potential that sensitivity/specificity of sural sparing from these is confounded by the presence of pre-existing compressive lesions.

The objective of this study is if sural sparing pattern specifically defined as absent/abnormal median and ulnar SNAP amplitude with normal sural SNAP amplitude (AMUNS) is sensitive and specific when compared with either absent/abnormal median and normal sural (AMNS) or absent/abnormal ulnar and normal sural (AUNS) for AIDP, CIDP and select non-diabetic axonopathies (AXPs) and diabetic neuropathies (DNs).

## Materials and Methods

Subjects for newly diagnosed DMN were retrospectively identified using the ICD-9 codes 357.81 for CIDP and 357.0 for AIDP over the period of 2001–2010. Charts were reviewed by study authors (Raghav Govindarajan and Spurthi Sunil Surpur) and only those patients who met the previously defined criteria for AIDP (2) and CIDP (3) were included. AIDP and CIDP variants were not included since the EDX findings in these are generally variable and would likely preclude meaningful analysis. All patients had to be 18 years and above at the time of the EDX study. The study was approved by local institutional review board.

All patients included had their first EDX study prior to the initiation of treatment done at our institution. None of DMN patients had known diabetes and/or any other known neuromuscular disorder. Patients who had evolved from AIDP to CIDP were included only in the AIDP group provided they met the defined criteria on initial presentation.

50 DN and 20 AXP patients over the period of 2009–2010 were selected as controls. DNs included were gradually progressive glove-stocking type, distal symmetric polyneuropathy. We chose DN as it is one of the most common causes of neuropathy and can have mixed demyelinating/axonal features. Of the AXP subset, we chose vasculitic neuropathy as the most common as it is a purely axonal lesion and the symmetric presentation of vasculitis can be a differential for DMN. Of the 20 AXP, 10 were vasculitis (5 non-systemic vasculitic neuropathy, 2 Churg–Strauss syndrome, and 3 vasculitis associated with connective tissue disorder). Two were alcoholic neuropathy, four were chemotherapy induced neuropathy, and four were nutritional—two due to severe vitamin B1 deficiency and two from vitamin B12 deficiency. If multiple studies were done, the first EDX study at the time of diagnosis was included in the analysis.

Sensory nerve conduction studies were done as per previously established protocol (12). Briefly, a pair of recording electrodes were placed in line over the nerve at an interelectrode distance of 3–4 cm with active electrode placed closest to stimulator. Ring electrodes were used for fingers. Electrode placement was consistent and reproducible and follows guidelines in standard references. Stimulation was performed with current increased from 0 mA usually in 3–5 mA increments until recoded sensory potential is maximized. Averaging was typically done for low amplitude sensory nerve potentials. Measurements for peak latency were made with the cursor on the midpoint of the first negative peak of evoked sensory response. The onset latency was measured from the stimulus to the initial negative deflection for biphasic and positive deflection for triphasic waves. Velocity was calculated using the onset latency. Techniques for recording amplitude and velocity were as per standard guidelines. Temperature of palm/hand, foot or other involved area was assessed using a Cooper non-contact infrared thermometer. Warming of the limb or area affected was performed as needed using heat packs placed in the microwave for 1 min. Unless otherwise noted, the hand temperature was monitored continuously and remained between 32 and 36°C and the foot temperature was maintained between 30 and 36°C during the performance of the nerve conduction. The amplifier settings were as below:

- Frequency: 20–2 k
- Gain: 10–20 uV/division
- Sweep speed: 2 ms/division
- Typical current strength: 5–30 mA for supramaximal stimulation

Standard values for the laboratory were sural amplitude >5 μV, median sensory amplitude (wrist) >20 μV, ulnar sensory amplitude >10 μV.

We defined sural sparing as normal sural SNAP amplitude bilaterally compared to two abnormal or absent upper limb SNAP amplitudes either on the same limb or the opposite limb. Since radial nerve examinations were infrequently performed as a part of the “peripheral neuropathy” work-up in our laboratory, we stipulated that both median and ulnar SNAP amplitudes were available for the purpose of our analysis. We used these strict criteria to get a more accurate estimate of sensitivity/specificity of sural sparing. Both median and ulnar nerves are commonly susceptible to compression and thus using two abnormal/absent upper limb SNAP amplitudes will reduce the likely hood of a false positive sural sparing from a preexisting compressive neuropathy.

### Statistical Analysis

Summary statistics were provided for demographic variables. Hypothesis testing for continuous variables was accomplished through *t*-tests. Main study categorical variables were analyzed with chi square initially comparing DMN with AXP and DN independently and then together as a control group. Odds ratios and conditional probabilities were calculated for statistically significant associations. A *p* value of <0.05 was considered statistically significant. The statistical software Statistical Package for the Social Sciences version 18.0 was used to process the data collected.

## Results

### Patient Demographics

A total of 50 AIDP and 82 CIDP cases were reviewed over the period of 2001–2010. Of these, AIDP cases not included were due to the lack of EDX studies or incomplete EDX data prior to treatment. Most of these cases were diagnosed by a combination of clinical and CSF studies. The CIDP cases not included in the analysis were those who were already diagnosed a few months or even years back and were on treatment. They were usually referred to us for second opinion by another physician. After excluding the above patients, we were left with 20 AIDP and 23 CIDP cases that clearly met the inclusion criteria as outlined. Median duration between symptoms onset and NCS for AIDP was 2 weeks.

Table 1 summarizes the demographic characteristics of both DMN and controls.

**Table 1:** Demographic characteriscs of study population.

	AIDP	AXP	CIDP	DN
**Male sex (%)**			
Mean	58	63	36	70
**Age (years)**			
Mean	47.7	59.1	58.7	67.2
95% CI ± 2SD	40.5–54.8	55.2–63.1	48.6–68.9	63.1–71.4
**Race—Caucasian (%)**			
Mean	53	47	91	63
95% CI ± 2SD	23–82	20–75	81–100	36–90

*AIDP, acute inflammatory demyelinating polyneuropathy; CIDP, chronic inflammatory demyelinating polyneuropathy; AXP, axonopathy; DN, diabetic neuropathy*.

Table 1 shows the demographic distribution of the study population, both DMNs and controls. The males were 58% in AIDP, 36% in CIDP, 63% in AXP, and 70% in DN. All the patients were more than 18 with mean ages being 47.7 in AIDP, 58.7 in CIDP, 59.1 in AXP, and 67.2 in DN. As depicted, there was male predominance in all the groups except CIDP (*p* < 0.05). We suspect this discrepancy was due to the fact that we have not included diabetic patients with CIDP.

### Distribution and Patterns of Sural Sparing

AMUNS was seen 13 patients (65%) with AIDP. AMNS was seen in 15 patients (76%) with AIDP. AUNS was seen in 14 patients (72%) with AIDP.

AMUNS was seen in nine patients (39%) with CIDP. AMNS was seen in 13 patients (56%) with CIDP. AUNS was seen in 11 patients (49%) with CIDP.

AMUNS was seen in two patients (10%) AXP and in three patients (6%) with DN. Two patients with AXP had AUNS, whereas three patients had AMNS. Three patients with DN had AUNS and three patients had AMNS.

**Figure 1:**
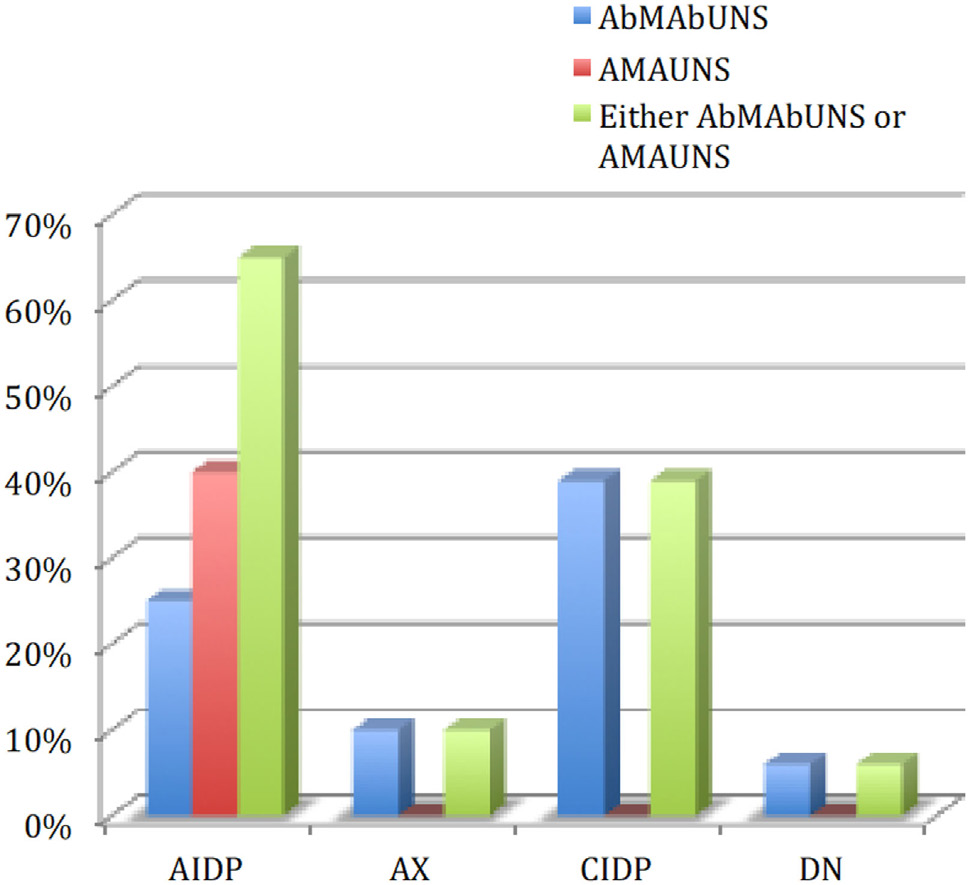
Distribution of “sural sparing” in the study population. AIDP, acute inflammatory demyelinating polyneuropathy; CIDP, chronic inflammatory demyelinating polyneuropathy; AXP, axonopathy; DN, diabetic neuropathy; AbM-AbU-NS, abnormal median abnormal ulnar and normal sural; AM-AU-NS, absent median absent ulnar and normal sural.

Figure 1 summarizes the % distribution of AMUNS among cases and controls.

Ten patients with DN also had abnormal median SNAP amplitude, abnormal ulnar SNAP amplitude and absent sural SNAP pattern. On the other hand, 13 AXP patients had abnormal median SNAP amplitude, abnormal ulnar SNAP amplitude, and either an absent or abnormal sural SNAP amplitude.

### Sensitivity/Specificity of Different Patterns of Sural Sparing

When AMUNS parameters are used for diagnosis of DMN (AIDP + CIDP), sensitivity is 51%, specificity is 92% (*p* < 0.001). Whereas the specificity of abnormal/absent median or ulnar with normal sural was 73% its sensitivity was 58%.

If a patient has sural sparing as defined by our strict criteria, he or she is more than 12 times more likely to have AIDP. Moreover, it should be noted that absent median SNAP amplitude absent ulnar SNAP amplitude with normal sural SNAP amplitude is seen only in the AIDP group. As a result, specificity and positive predictive value of this pattern is 100%. Individuals with this pattern are more than 127 (*p* < 0.001) times more likely to have AIDP than the controls.

## Discussion

Previous studies have included only a single abnormal upper limb SNAP—for example, median nerve (8, 9) in the definition of sural sparing. However, either the median or ulnar nerve may be commonly involved by an entrapment neuropathy. By including both nerves in the definition rubric, we have tried to reduce the likelihood of having false positive sural sparing patterns. In our study, the addition of both median and ulnar (especially absent median and ulnar with normal sural SNAP amplitude) reduced the sensitivity with significantly improved specificity when compared with inclusion of either median or ulnar in the AIDP cohort but did not have significant impact on the sensitivity or specificity of the CIDP cohort. The sensitivity of sural sparing was low and comparable to previous studies (7–11) which might limit its isolated clinical application. The low sensitivity might be due to abnormality of sural SNAP amplitude in the elderly, obese, in patients with preexisting polyneuropathy or even with pedal edema.

The diagnosis of AIDP remains clinical in many cases and the addition of electrodiagnostic study might be to rule out mimickers. Thus, a stricter criterion of sural sparing with improved specificity might be beneficial to confirm AIDP from other mimickers but may not be that beneficial in CIDP.

The lower percentage of sural sparing seen in CIDP might be due to the insidious nature of this condition, older age of presentation where there is an increased likelihood that the sural SNAP amplitudes will be abnormal and even the fact that we have not screened our CIDP patients for glucose intolerance. The florid form of sural sparing (absent median SNAP amplitude, absent ulnar SNAP amplitude with normal sural SNAP amplitude) was also not seen in CIDP. The slow evolving course of CIDP and the fact that we have not included those who have evolved from AIDP might have accounted for this.

### Limitations

Being a retrospective study there was a lack of uniform testing procedure especially with regard to the nerves tested which is what precluded the inclusion of radial SNAP amplitude in the rubric defining sural sparing.

We have defined sural sparing as requiring both abnormal/absent median and ulnar SNAP amplitudes to avoid inadvertent inclusion of patients with unrelated distal median and ulnar nerve entrapment injury. However, both ulnar and median nerves are susceptible to random multifocal demyelination with conduction block and phase cancelation especially at the entrapment sites thus potentially confounding the results (13). Some authors have suggested a proxy of the SNAP ratio: a sensory ratio whereby the sum of sural and median SNAPs is divided by the sum of median and ulnar SNAPs as a way of improving the diagnosis (7).

We have not analyzed quantitative sensory electrodiagnostic parameters, such as sensory nerve action potential amplitudes, durations, sensory nerve conduction velocities or even sensory nerve conduction blocks. Some authors have found these to be highly specific and increased the sensitivity of sural sparing (11, 14).

## Conclusion

Sural sparing is highly specific but not sensitive when compared with either AMNS or AUNS in AIDP but does not add to sensitivity or specificity in CIDP.

## Ethics Statement

The study was approved by University of Missouri Institutional Review Board. Since the study involved a retrospective deidentified chart review, the study was granted an exemption for getting informed consent from patients.

## Author Contributions

SS: manuscript editing. RG: principle author.

## Conflict of Interest Statement

The authors declare that the research was conducted in the absence of any commercial or financial relationships that could be construed as a potential conflict of interest. The reviewer HS and RB and handling Editor declared their shared affiliation.

## Abbreviations

AIDP, acute inflammatory demyelinating polyneuropathy; CIDP, chronic inflammatory demyelinating polyneuropathy; SNAP, sensory nerve action potential; DMNs, demyelinating neuropathies; AXPs, axonopathies; N, normal; S, sural; M, median; U, ulnar; AMUNS, absent/abnormal median and ulnar SNAP amplitude with normal sural SNAP amplitude; AMNS, absent/abnormal median with normal sural; AUNS, absent/abnormal ulnar with normal sural.
